# To the Public

**Published:** 1819-10-01

**Authors:** 


### TO THE PUBLIC.

If we trace the History of Arts, Sciences, and every human institution, we shall perceive an endless series of changes and innovations marking their progressive advances towards perfection or maturity. It is, in truth, the province of wisdom and philosophy to profit by experience, and make each day a critique on the past; while it is the character of folly and arrogance to vacillate without cause, change without object, or persevere against conviction. The "tenax propositi" is a dangerous maxim in Literature, as well as in Politics. Let not then the *modifications* which this Journal has exhibited, be hastily ascribed to caprice or instability. It will be found, on a strict examination, that each innovation has been an *improvement* on the preceding form, and an addition of labour and responsibility to the conductors.

None but the *latter*, indeed, can know or appreciate *all* the reasons which influence their conduct—none surely can have so deep an interest in the welfare of the work, as those whose property is embarked in it, and whose reputation is involved in its final success.

It has been found, then, impracticable to keep pace with the progress of Medical Science in the Review Department, and, at the same time allot, in every Number, a sufficient space for Original Communications and Foreign Translations. The force and the truth of this observation are amply illustrated in every Medical Journal of the United Kingdom.*

As we have received, from every quarter, testimonies of the most unequivocal approbation of our Critical Analyses, and solicitations to carry them to the utmost extent; so, in compliance with these wishes, the Medico-Chirurgical Journal has assumed, for the present at least, the character of an Analytical Review.†

The widely extended circle of professional Friends and Correspondents with whom the Editor has the honour of being connected, secures to him, at all times, an unlimited auxiliary force, which he hopes will be directed with energy and effect.

The paramount objects of the Medico-Chirurgical Journal may be succinctly stated as follows:—

First, To generate in the minds of medical students an early turn for observation; a habit of reflection; and a taste for medical literature.

Secondly, To bring within the reach of those classes of medical

---

* Upwards of *one hundred* important medical works have issued from the press during the last twelve months. Whether an adequate Analytical Review of these has appeared in Medical Journals, we leave to the Reader. In this Journal, which devotes so much space to the Review Department, justice has not been rendered to *one half* of the works published. What then is to be done?

† The numerous *periodical* Transactions of the day, independently of the Monthly Journals, open a wide door for *Original* Communications, and imperiously demad an exclusive Review.

society who may not have the leisure to peruse, the means to purchase, or the opportunity to obtain, the various works that issue from the press, a *comprehensive, yet concentrated analysis* of all important medical publications, enlivened with practical, rather than with *biblical* criticism, which last is more calculated to indulge the vanity of the writer, than improve the understanding of the reader.*

THIRDLY, To enable those who *have* the means and the desire of forming a selection from the current of medical literature to *judge for themselves*, without the previous labour and expence of perusing and purchasing many *useless* publications.†

To the GENERAL PRACTITIONERS of Great Britain, and to the Medical Officers of the *Army*, the *Navy*, and the *Colonies*, a Journal, well conducted on the above-mentioned principles, must prove an invaluable acquisition; and to the patronage of *these*, in particular, it is dedicated. "As for those," (to use the words of Huxham), "who will neither read nor reason, but practise by rote, and prescribe at a venture, we seriously advise then to peruse the SIXTH COMMANDMENT."

To our numerous and able Correspondents, in the *Original* Department, we return most grateful thanks for past favours, and still solicit from them such practical hints, observations, remarks, and *concise* cases as their professional rounds may bring to light. These will be incorporated, or introduced in the most appropriate situations of the *Review*, and *always with strict reference to the sources whence they are derived*, unless the anonymous veil be ordered to continue; as, for example, at page 277 of the present Number. In this way circulation may be given to a vast body of useful matter, in a comparatively small space, and with quite as much honour and publicity to the communicants, as though their papers were dilated to formal, or formidable lengths.‡

We need say no more. A specimen of the work is now before that Public which is well able to decide on its merits. It would be equally useless and degrading to solicit indulgence, or to hope for patronage, without returning the *quid pro quo*. This we have endeavoured to do; and if we have failed in the attempt, the Literary Mart is still open to the Public. It is by liberal competition not sordid monopoly that we wish to stand or fall. "Qui stadium currit, eniti et contendere debet quàm maximè possit, ut vincat: supplantare eum, quicum certet, aut manu depellere, nullo modo debet." *Cic. Offic.*

---

* No purely *Critical Journal* of Medicine ever did or ever will maintain its ground in this country; whereas, *Analytical* Reviews have ever been the favourites of all ranks of Medical Society.

† This plan will not exclude *Reviews* of good Foreign Publications; on the contrary, there will, in general, appear an Analytical Review of some *important* foreign work in each Number of the Journal. The article of this description in our next, will be found of great value.

‡ Various examples of this plan will be seen in the ensuing Number; in the present we have been obliged to review *exclusively*, in order to bring up arrears, as much as possible; and still we are in debt.

